# Perinatal depression and resilience in Polish women during the war-inflation crisis

**DOI:** 10.1192/j.eurpsy.2025.2390

**Published:** 2025-08-26

**Authors:** E. Barszcz, Z. Blaszczyk, A. Wyrzychowski, M. Plewka, O. Gawlik-Kotelnicka

**Affiliations:** 1 Faculty of Medicine, Medical University of Łódź; 2 Department of Clinical Psychology and Psychopathology, University of Łódź; 3 Department of Affective and Psychotic Disorders, Medical University of Łódź, Łódź, Poland

## Abstract

**Introduction:**

The ongoing armed conflict in Ukraine has significantly destabilized Europe’s geopolitical and economic situation, leading to a war-inflation crisis that may substantially affect the mental health of women during the perinatal period.

**Objectives:**

Our primary objective was to investigate the prevalence of depressive symptoms and assess the role of resilience as a protective factor against anxiety and depression in Polish perinatal women during the war-inflation crisis. We analyzed the percentage of women experiencing anxiety related to childbirth and the war-economic crisis, as well as their relationship with perinatal depression. The study also aimed to identify risk factors for perinatal depression and various types of anxiety, including those related to childbirth, war, and the global situation.

**Methods:**

152 women participated in three online surveys – two conducted during pregnancy and one after childbirth. To evaluate mental well-being and the intensity of depressive and anxiety symptoms, we utilized the Edinburgh Postnatal Depression Scale (EPDS), Beck Depression Inventory (BDI-2), Labour Anxiety Questionnaire (LAQ), along with research team-developed questionnaires assessing anxiety related to the war (WAQ) and global situation (GSAQ). Resilience was assessed using the Resilience Measure Questionnaire (KOP-26).

**Results:**

About 32.2% of perinatal women were diagnosed with depression based on the EPDS scale with a cutoff of ≥14. Nearly 70% scored 14 or higher on the LAQ scale, indicating a significant rise in labour-related anxiety. Additionally, 24.3% experienced high levels of anxiety due to the war, while 25% faced severe anxiety related to the global situation. The Kruskal-Wallis analysis, with resilience as the independent variable, revealed statistically significant differences in the distribution of depression (F=28.302; df=2; p<0.001) and global situation anxiety (F=7.183; df=2; p<0.028) variables between the groups. Post hoc analysis showed differences in the severity of depressive symptoms and global situation anxiety between the low and high resilience groups. The correlation heat map between psychometric scores at the start of the study is presented in Table 1.

**Table:** 

	EPDS	LAQ	WAQ	GSAQ
**EPDS**				
**LAQ**	r=.53 p<.001			
**WAQ**	r=.10 p=.205	r=.21 p=.008		
**GSAQ**	r=.33 p<.001	r=.40 p<.001	r=.27 p=.001

**Conclusions:**

The prevalence of depressive disorders among women in the perinatal period may increase during crises caused by war and inflation, compared to periods of geopolitical and economic stability. This is a strong argument for improving the screening system for perinatal depression in Poland. A lower level of resilience during pregnancy may be a significant predictor of increased severity of depressive symptoms and higher levels of anxiety related to global situation among the perinatal population.

**Disclosure of Interest:**

None Declared

